# Acetazolamide-responsive myotonia with a novel Ile239Thr mutation in *SCN4A* gene: a case report

**DOI:** 10.1097/MS9.0000000000001673

**Published:** 2024-01-03

**Authors:** Jashpal Yadav, Ritesh Barnwal, Sujit Kumar Mandal, Bina Prajapati

**Affiliations:** a Dhakdhai Primary Health Care Center, Rupandehi; b Department of Neurology, Kanti children’s hospital; c Ministry of Health and Population, Kathmandu, Nepal

**Keywords:** acetazolamide, case report, dive-bomber sound, SCN4A gene, sodium channel myotonia

## Abstract

**Introduction and importance:**

Sodium channel myotonia (SCM) belongs to the group of sodium channelopathies with mutations involving *SCN4A* gene. The main feature of sodium channel myotonia is pure myotonia without episodes of weakness or paralysis. One of the sodium channel myotonia has been classified as acetazolamide-responsive myotonia because of the effectiveness of acetazolamide as an antimyotonic drug.

**Case presentation:**

The child presented with generalized muscle hypertrophy and stiffness involving arms, thighs, calves, chest, and back muscles with unusually prominent trapezius muscle. The parents described the warm-up phenomenon as an improvement in stiffness as the day passes and with repetitive action. Percussion myotonia was illustrated in the thenar eminence and trapezius muscle. Characteristic ‘dive-bomber’ sound was present in electromyography, and whole-exome sequencing revealed a novel Ile239Thr mutation in the *SCN4A* gene. Acetazolamide was prescribed for the condition, and regular follow-up shows an excellent clinical response.

**Clinical discussion:**

This case presents a pure myotonic phenotype without episodes of weakness or paralysis. Generalized myotonia with muscle hypertrophy and demonstrating warm-up phenomenon resembles myotonia congenita (a chloride channelopathy). However, genetic analysis revealed a novel Ile239Thr mutation involving *SCN4A* gene indicating this case to be a sodium channelopathy.

**Conclusion:**

This case limelight sodium channel myotonia with a novel Ile239Thr mutation in *SCN4A* gene that phenotypically resembles myotonia congenita but genetically belongs to sodium channelopathy highlighting the poor correlation between genotypes and phenotypes in non-dystrophic myotonia. Acetazolamide can be a safe and cost-effective antimyotonic drug in sodium channel myotonia.

## Introduction

Highlights

- A patient with symptoms strongly indicating myotonia congenita was found to have a new Ile239Thr mutation in the SCN4A gene, confirming the sodium channelopathy nature of the condition.
- Highlights challenges in linking genotypes to phenotypes in non-dystrophic myotonia.
- Acetazolamide proves a safe and cost-effective treatment for this sodium channel myotonia case.
- Some cases of sodium channel myotonia, including the one presented, are classified as acetazolamide-responsive myotonia, emphasizing the efficacy of acetazolamide as an antimyotonic drug.

Sodium channel myotonia (SCM) belongs to the group of sodium channelopathies presenting with myotonia. Myotonia refers to impaired relaxation of skeletal muscles after voluntary contraction or percussion. SCM exhibit autosomal dominant inheritance with gain of function mutation involving *SCN4A* gene which encodes for alpha subunit of voltage-gated Na^+^ channel (Nav1.4)^1^. These Na^+^ channels are found abundantly in skeletal muscles and are responsible for the generation of action potential leading to the normal contraction of the muscle fibres. After the depolarization of sarcolemma, these voltage-gated sodium channels become rapidly inactivated (fast inactivation) and return to their resting conformation until the repolarization has occurred. Repolarization occurs via opening of K^+^ channels and these potassium ions get accumulated in T-tubules and are enough to generate after-depolarization which is counter-balanced by chloride channels^2^. However, gain of function mutation involving Nav1.4 channels result in the impairment of inactivation or enhanced activation of these channels; a common underlying defect of all sodium channelopathies. Impaired inactivation can be due to either delayed inactivation of Nav1.4 channels causing hyperexcitability of sarcolemma leading to myotonia which is the main feature of SCM or incomplete inactivation resulting in sustained depolarization of sarcolemma leading to weakness as seen in Hyperkalemic periodic paralysis^3^.

Sodium channel myotonia represents purely myotonic phenotypes with absence of episodic weakness. Onset of first symptom is variable occurring within the first decade or may presents late during adolescence^3^. The most frequently affected muscles are muscles of eyelids, extraocular muscles, jaw, upper limb and lower limb with or without hypertrophy. Both the warm-up phenomenon (repetitive contraction improves myotonia) and paradoxical worsening of myotonia can be seen. The clinical features may overlap with paramyotonia congenita and myotonia congenita^4,5^.

SCM is classified into Acetazolamide-responsive myotonia, myotonia fluctuans, myotonia permanens. These three groups share common features of myotonia aggravated with potassium ingestion and insensitivity to cold, sometimes referred together as potassium aggravated myotonia^6^. In acetazolamide-responsive myotonia, there is generalized myotonia exacerbated by potassium ingestion without periodic weakness or paralysis. Muscle hypertrophy may be seen. Myotonia is often painful and demonstrate remarkable improvement with acetazolamide^5^. In myotonia fluctuans, patients present with fluctuating myotonia which are characteristically of delayed onset (10–30 min) after exercise unlike paramyotonia congenita. More severe and persistent myotonia affecting breathing and swallowing is seen in myotonia permanens^3,6^.

Out of all antimyotonic drugs used in non-dystrophic myotonia; mexiletine has been the most effective, and other medications such lamotrigine, acetazolamide, carbamazepine, flecainide, ranolazine has been used with variable degree of success. Mexiletine is a class Ib antiarrhythmic drug which works on the voltage dependent sodium channels in both skeletal muscles (Nav1.4) and cardiac muscles (Nav1.5). It has been used as a first line antimyotonic drug in non-dystrophic myotonias including sodium channel myotonia with level I evidence. It works through enhancement of fast inactivation of Na^+^ channels. Acetazolamide also has been found to be effective in improving the myotonia especially in sodium channel myotonia and hyperPP. Acetazolamide increases chloride conductance through direct effect on skeletal muscle chloride channels due to intracellular acidification^2^.

This case phenotypically resembles myotonia congenita but genetically with Ile239Thr mutation involving the *SCN4A* gene. This case report has been reported in line with SCARE guidelines^7^.

## Case presentation

**Figure 1:**
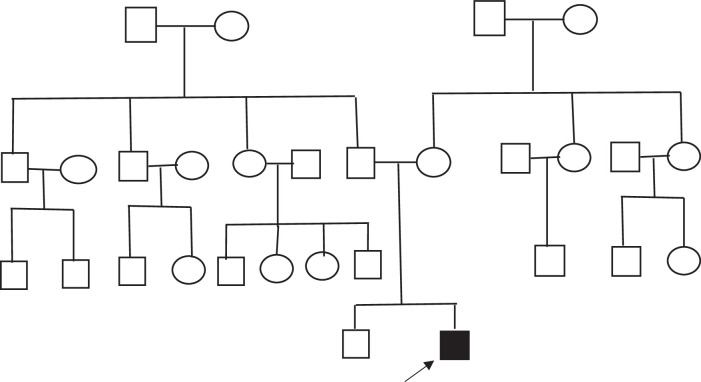
Pedigree of the family. In the diagram: Black-filled symbols represent family members who are affected by the condition. An arrow points to the “index case,” which is the individual initially identified as having the condition or the one through whom the family’s medical history is being studied. Notably, no other family members exhibit any signs or symptoms of the disease. Genetic testing was limited to the index case due to financial limitations. However, it’s essential to note that comprehensive understanding of the inheritance pattern within the family might require genetic testing for additional members.

Two years and ten months’ male child presented to the neurology department with complaints of swelling of different body parts since the boy was 5 months of age. According to his mother, he was apparently asymptomatic till five months of age when his mother started noticing swelling in the upper back and front chest, which was insidious, progressive, and non-painful. By one year of age, the swelling had progressed to involve bilateral upper and lower limbs, mainly over the arm, thigh, and calf region. The mother also noticed that the child was clumsy. However, the child achieved his developmental milestones as per his age. The child had difficulty standing up from a sitting position, abnormal and slow walking, with frequent falling while walking. The child was characteristically slow in the morning after getting up from bed, and the activity improved as the day passed. The child was born by normal vaginal delivery at term gestation without any complications during delivery and cried immediately after birth. He is the second child of non-consanguineous parents, with an older brother of 11 years. There is no history of a similar illness in the family (Fig. 1).

**Figure 2:**
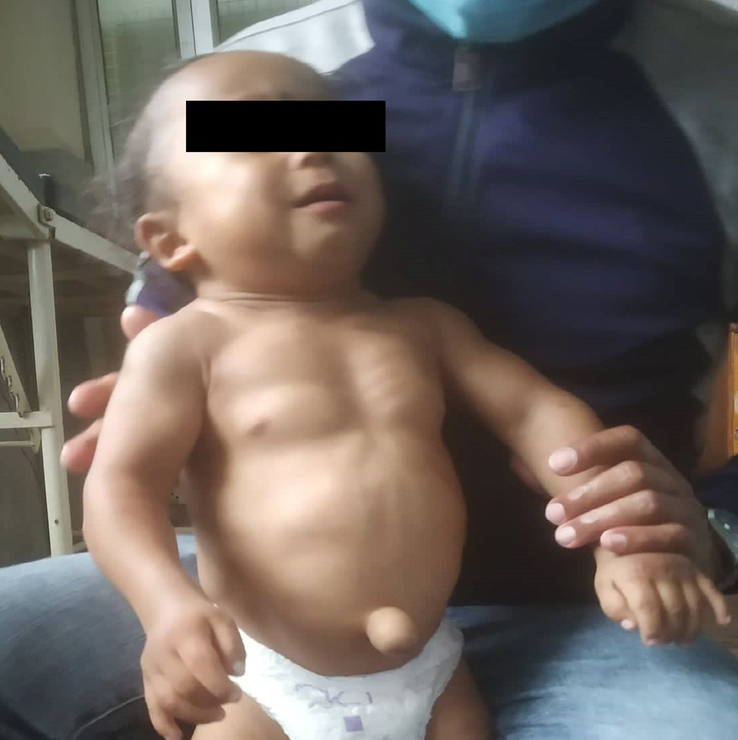
Showing prominent muscles of cheeks, chest, abdomen, arms, and thigh with umbilical hernia.

**Figure 3:**
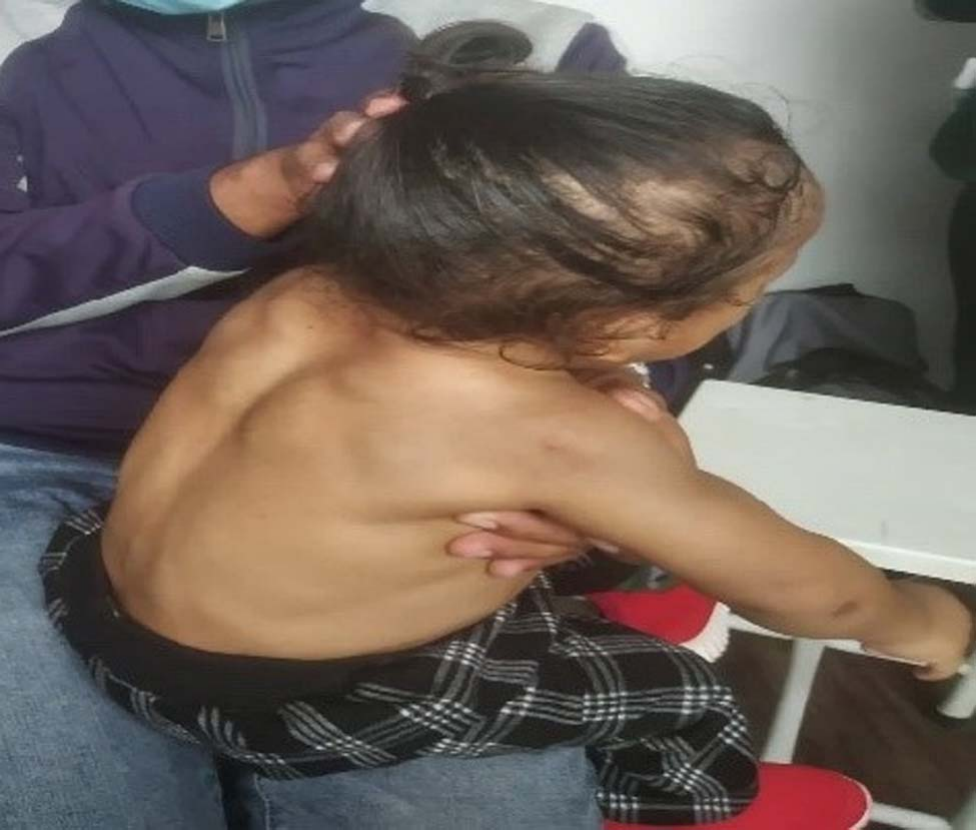
Showing unusually hypertrophied trapezius muscle and prominent muscles over the scapula.

On general examination, the patient had prominent muscles of cheeks, chest, abdomen, scapular region, arms, thighs, and calves with unusually hypertrophied trapezius muscle (Figs. 2 and 3). There was delayed relaxation of hands after making a fist, and when stroked with a knee hammer, there was a tonic contraction of the thenar muscles and trapezius. The rest of the neurological examination was grossly normal. Except for reducible umbilical hernia, abdominal, respiratory, and cardiovascular systems were intact.

Complete blood count, HbA1c, thyroid function test, vitamin D level, creatine kinase, ECG and echocardiography, and brain and spinal cord MRI were normal. The needle electromyography study on left deltoid and left quadriceps muscle revealed a “dive-bomber” sound characteristic of myotonia with normal insertional activity, no fibrillations, fasciculation and positive sharp waves, normal motor unit action potential (MUAP) morphology and recruitment pattern. With these clinical findings, myotonia congenita was suspected initially and planned for genetic analysis.

Whole-exome sequencing was conducted using advanced next-generation sequencing technology. The coverage rates for the target bases were 99.32% at 1×, 99.05% at 10×, 96.10% at 20×, and 88.80% at 30×. During the sequencing process, a heterozygous missense variant (NM_000334.4: c.716T>C) was identified in the *SCN4A* gene. This variant results in the substitution of Isoleucine with Threonine at position 239 in the protein (p.(Ile239Thr)). The inheritance pattern was determined to be autosomal dominant, and the variant was classified as likely pathogenic. Considering the history, clinical examination findings, myotonic discharges on EMG report and the whole-exome sequencing report, the final diagnosis of sodium channel myotonia was made, and the patient was prescribed acetazolamide 125 mg orally twice a day. The regular follow-up every 3 months has shown an excellent clinical response, and the parents are also happy with the improvement in the child’s condition. Annual renal ultrasound scan and renal function test have not shown any adverse events to date (Table 1).

**Table 1:** The baseline and follow-up renal function tests, including the levels of sodium (Na+) and potassium (K+)

	Urea (mg/dl)	Creatinine (mg/dl)	Na^+^ (mmol/l)	K^+^ (mmol/l)
Baseline	26	0.5	140	4.1
Follow-up (annual)	29	0.4	136	3.8

## Discussion

**Figure 4:**
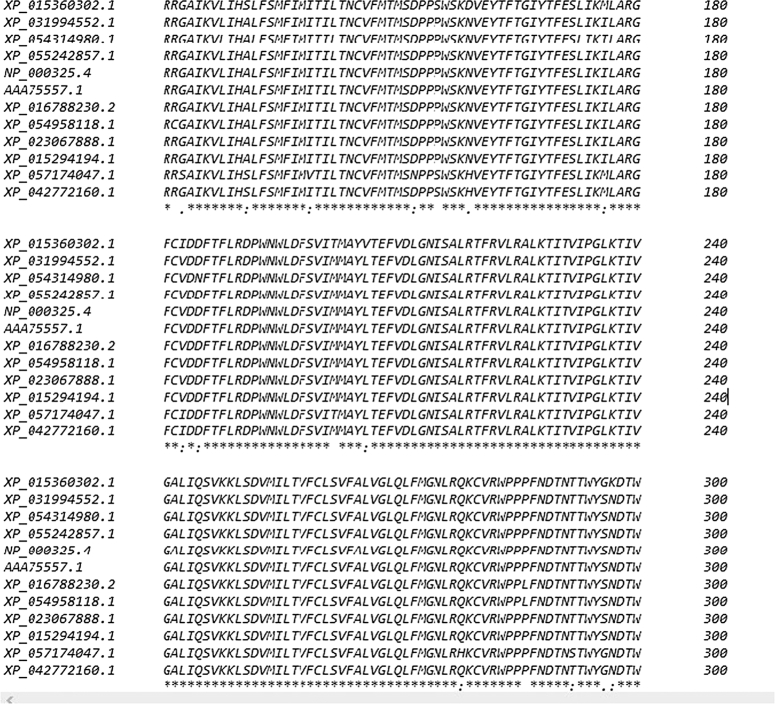
Clustal 0 (1.2.4) multiple sequence alignment is presented, showing a detailed comparison of sequences from different vertebrate species. The specific focus of the figure is on position 239 within these sequences. The information conveyed indicates that the amino acid isoleucine, found at position 239, is remarkably preserved and consistent across a diverse range of vertebrate species. This high conservation suggests that isoleucine at this particular position likely plays a crucial role in the biological function or structure of the protein under study.

The early onset of generalized muscle hypertrophy and myotonia affecting the upper and lower limbs, chest, back, and abdominal muscles results in an unusual gait and frequent falls. Stiffness improves with repetitive movements, displaying a warm-up phenomenon. These symptoms are more indicative of myotonia congenita^6^. However, genetic analysis revealed a mutation in the *SCN4A* gene, indicating sodium channelopathy as the underlying condition. The absence of weakness or paralysis suggests a pure myotonic phenotype in this case. Painful myotonia has been documented in sodium channelopathies, especially in cases responsive to acetazolamide^5,8^. Interestingly, our patient did not report painful myotonia, possibly due to their young age or the myotonia might not have been painful after all. Additionally, there were no signs of paramyotonia congenita, such as worsening stiffness with exercise and exposure to cold. The electromyography findings were consistent with myotonic discharges, aligning with the majority of non-dystrophic myotonia cases. Around 83 mutations have been documented in the *SCN4A* gene, with approximately 65 linked to myotonia^8^. In our study, we discovered a new genetic variation (NM_000334.4: c.716T>C) in the *SCN4A* gene, leading to the amino acid substitution Isoleucine to Threonine at position 239 in the *SCN4A* protein. This particular variant was not found in widely used databases such as dbSNP and gnomAD, indicating its novelty and absence from existing genetic datasets. To assess its significance, we conducted a multiple sequence alignment of the *SCN4A* protein (RefSeq accession number NP_000325.4) using Clustal Omega^9^. The alignment revealed that Ile239 is highly conserved across different vertebrates (as shown in Fig. 4), highlighting its probable functional importance for Nav1.4 channels.

The Ile239 Thr change in the *SCN4A* protein was predicted to be deleterious using bioinformatics tools SIFT and Polyphen-2^10,11^. This mutation likely impairs the inactivation of Nav1.4 channels after the sarcolemma has depolarized. This disruption explains the observed myotonic discharges in electromyography (EMG) and the clinical myotonia in affected individuals.

It’s important to note that whole-exome sequencing, while valuable, might not detect certain variants like intronic variations, repeat expansions, copy number variations, or chromosomal rearrangements.

The patient’s family lacked similar symptoms, suggesting a de novo origin of the mutation, although genetic testing was not conducted due to financial constraints.

Mexiletine is the most effective antimyotonic drug in non-dystrophic myotonias, including sodium channel myotonia. Other medications like lamotrigine, acetazolamide, carbamazepine, flecainide, and ranolazine show variable success. Acetazolamide, especially in sodium channel myotonia and hyperPP, improves myotonia by increasing chloride conductance in skeletal muscle^2,4^. Considering the cost and potential pro-arrhythmogenic effects of mexiletine, and the efficacy of acetazolamide in improving myotonia in SCM, acetazolamide was chosen as the antimyotonic drug^12,13^. However, the treatment carries risks of nephrolithiasis, electrolyte imbalances, and paraesthesia. Therefore, baseline ultrasound scans, renal function tests with electrolytes, and annual ultrasound monitoring for nephrolithiasis are strongly recommended. Regular renal function tests and electrolyte monitoring after each dose adjustment are also advised^2^. Lifestyle changes that can aid patients with myotonia include avoiding abrupt movements and gradually warming up before activities. Individuals with paradoxical myotonia (PMC, HyperPP, and SCM) find relief through rest, suitable clothing, and indoor activities to minimize cold-induced exacerbation. While most myotonia patients are not impacted by dietary alterations, those with sodium channel mutations, including HyperPP, might be sensitive to a diet high in potassium^2,4^.

## Conclusion

This case sheds light on sodium channel myotonia due to a novel Ile239Thr mutation in the *SCN4A* gene. Despite its clinical similarity to myotonia congenita, a disorder linked to chloride channelopathy, the genetic analysis categorically places it within sodium channelopathy. This discordance underscores the limited understanding of the relationship between genetic variations and observed symptoms in non-dystrophic myotonia. Additionally, the effective use of acetazolamide in this sodium channel myotonia case emphasizes its potential as a safe and economical antimyotonic treatment, offering hope for managing this condition. This case challenges our understanding of these disorders, highlighting the need for further research to bridge the gap between genetic variability and clinical presentation in myotonic disorders.

## Ethical approval

None.

## Consent

Written informed consent was obtained from the patient’s parents/legal guardian for the publication of this case report and accompanying images. A copy of the written consent is available for the review by the Editor-in-Chief of this journal on request.

## Source of funding

None.

## Author contribution

J.Y. was involved in conceptualization, validation, and drafting of the manuscript, as well as reviewing and editing it. R.B. contributed to the investigation, data collection, and manuscript editing. S.K.M. assisted in drafting the manuscript and provided final review and editing. B.P. conducted the investigation, provided supervision and guidance, and also participated in editing the manuscript.

## Conflicts of interest disclosure

None.

## Research registration unique identifying number (UIN)

None.

## Guarantor

Jashpal Yadav.

## Provenance and peer review

Not commissioned, externally peer-reviewed.

## Data availability statement

Available upon reasonable request.
